# Educational attainment among survivors of childhood cancer: a population-based cohort study in Denmark

**DOI:** 10.1038/sj.bjc.6602085

**Published:** 2004-08-03

**Authors:** S V Koch, A M T Kejs, G Engholm, C Johansen, K Schmiegelow

**Affiliations:** 1 Department of Psychosocial Cancer Research, Institute of Cancer Epidemiology, Danish Cancer Society, Strandboulevarden 49, DK-2100 Copenhagen, Denmark; 2 Section of Paediatric Haematology and Oncology, Paediatric Clinic II, Juliane Marie Centre, University Hospital, Blegdamsvej 9, DK-2100 Copenhagen, Denmark; 3 National Institute of Public Health, Svanemøllevej 25, DK-2100 Copenhagen, Denmark; 4 Department of Cancer Prevention and Documentation, Danish Cancer Society, Strandboulevarden 49, DK-2100 Copenhagen, Denmark

**Keywords:** childhood cancer, population-based cohort study, registry, Cox regression, educational attainment, central nervous system tumour

## Abstract

We identified 2384 patients in the Danish Cancer Register in whom cancer had been diagnosed in 1960–1996 before they reached the age of 20 and compared them with 53 143 sex- and age-matched controls identified from the Register of Population Statistics. Complete education records and demographic and socioeconomic information for the period 1980–2000 were obtained for both cohorts from Statistics Denmark. The rate ratio (RR) for educational attainment was estimated by discrete-time Cox regression analyses. An overall reduction in attaining basic education was found (RR, 0.90; 95% confidence interval, 0.83–0.96). Female survivors of central nervous system (CNS) tumours showed the largest educational deficit (RR, 0.55; 95% confidence interval, 0.37–0.82). Non-CNS tumour survivors attained education as controls at most levels. When the analyses were conditioned on completion of youth education, further educational attainment was not reduced for any group of survivors. These findings confirm that only survivors of CNS tumours in childhood experience significant educational deficits. The deficit was mainly seen among persons whose tumour was diagnosed before they reached the level of secondary education.

Improvements in the treatment of cancer in children and adolescents since the 1970s mean that today more than two-thirds of childhood cancer patients survive (de Nully *et al*, 1995; Berrino *et al*, 2001). The psychosocial implications of living after cancer have therefore increasing relevance. Educational level is an important indicator of a person's capabilities and motivation; it is also an indicator of socioeconomic status and a predictor of health outcomes (Lynch and Kaplan, 2000).

Reintegration into daily life at school is one of the main challenges after diagnosis and treatment of cancer because of long absences from school (Charlton *et al*, 1991). Cancer survivors may face emotional difficulties, lack of energy, adjustment problems, social isolation, and restrictions on physical activity (Mancini *et al*, 1989; Adamoli *et al*, 1997; Glaser *et al*, 1997; Eiser and Vance, 2002). Treatment with cranial radiation therapy can affect academic performance by significantly decreasing the IQ or by more subtle neuropsychological effects (Jankovic *et al*, 1994; Dongen-Melman *et al*, 1997; Reimers *et al*, 2003).

Some previous studies found that the overall educational attainment of survivors was equivalent to that of comparison groups, except for survivors of central nervous system (CNS) tumours and of acute lymphoblastic leukaemia who had received cranial radiotherapy (Kelaghan *et al*, 1988; Haupt *et al*, 1994; Pastore *et al*, 2001). Other studies concluded that survivors of childhood cancers more generally had significant deficits in educational outcome (Teta *et al*, 1986; Langeveld *et al*, 2003; Mitby *et al*, 2003). Although well conducted, most previous studies were subject to incomplete participation, with rates as low as 61%, and suboptimal comparison groups, including siblings (Teta *et al*, 1986; Kelaghan *et al*, 1988; Haupt *et al*, 1994; Mitby *et al*, 2003) and population values (Pastore *et al*, 2001); healthy controls were used in only one study (Langeveld *et al*, 2003).

To explore educational attainment after childhood cancer we established a nationwide, population-based cohort of persons who had had cancer in childhood or adolescence and compared them with controls sampled from the general population (Koch *et al*, 2004).

## MATERIALS AND METHODS

### Study population

We identified all cancer survivors: (i) in whom the disease was diagnosed in Denmark between 1960 and 1996 before they reached the age of 20 years; (ii) who had had a benign CNS tumour or a cancer other than myelodysplastic syndrome; (iii) who were born in Demark in 1960–80, to ensure sufficient data quality and maximal follow-up time; (iv) who had at least one parent who was a citizen of Denmark, Finland, Iceland, Norway or Sweden, to reduce cultural heterogeneity; (v) who were 13 years of age or older at the start of follow-up; and (vi) who were alive at least once on 31 December in the period of follow-up from 1980 to 2000, where the socioeconomic data were available.

The patients were identified in the Danish Cancer Register, which covers virtually all incident cases of cancer that have occurred in Denmark since 1943 and has been described in detail elsewhere (Storm *et al*, 1997). From this Register, we obtained information on diagnosis, tumour type, date of diagnosis, tumour location, vital status and the unique personal identification number that has been assigned to all residents in Denmark since 1 April 1968. The personal identification number includes sex and date of birth and permits accurate linkage of information between registers. Diagnoses were classified according to the International Agency for Research on Cancer classification of childhood cancers (Birch and Marsden, 1987). No reliable treatment variables were available from the Danish Cancer Register for this study.

A randomly sampled control cohort, frequency matched on sex and date of birth, was identified in the Register of Population Statistics (Eurostat/Statistics Denmark, 1995), which contains basic demographic information on the total Danish population. The information in this Register is derived from the Central Population Register, and it is the source of personal and population statistics for other registers (Eurostat/Statistics Denmark, 1995; Thygesen, 1995). The parents and siblings of all the study subjects were also identified through this Register.

Of the sampled controls, 0.2% were excluded because they were not registered at 31 December in 1980–2000. A further 0.1% of the controls were excluded from the control cohort because they were also identified in the cancer cohort. A total of 2384 individuals who had had childhood cancer and 53 143 controls were eligible for analysis, contributing 30 792 and 803 644 person-years, respectively.

### Estimation of educational attainment

Information on all persons in the two cohorts and their parents was linked to the Integrated Database for Labour Market Research, which contains annually (by 31 December) updated information on the total Danish population from 1980 onwards (Statistics Denmark, 1991). From this Database, we obtained demographic and socioeconomic variables for the end of each year between 1980 and 2000, including type and level of education, family structure, labour market participation and income. The Database holds information only on the level of education completed and not on graduation, marks or use of special education services.

All education in Denmark is free of charge, and the Danish State provides economic support during studies to facilitate the education of persons of lower socioeconomic status (SU, 2004). The levels of education used in this study were those of the Danish educational system, shown in Table 1

**Table 1:** Danish educational system

	**Education**			**ISCED code**
**Age (years)**	**Level**	**Subcategory**	**Description**	
6	Preschool (optional)			0
7–16	Basic	Years 1–9	Compulsory	1 (years 1–7)
		Year 10	Optional	2 (years 8–10)
17–19	Youth	Upper secondary school	Traditional, or vocational, higher technical or higher commercial examination	3
		Vocational training	Leads to jobs like: skilled craftsman, service function in business and trade, assistant social worker, assistant nurse, waiter, cook	3
20–24	Higher education	Short cycle (2 years)	Leads to jobs like: programmer, bachelor in commerce, laboratory technician, caterer, policeman	4
		Medium cycle (4 years)	Leads to jobs like: kindergarten teacher, basic school teacher, nurse, journalist, social worker, midwife, engineer	5
		Long cycle (3–5 years)	Leads to masters or jobs in: language, literature, dentistry, medicine, social science, law, theology, natural sciences	5
25–27		PhD	Leads to jobs in: same as 5	6

Modified from Statistical Yearbook 2002 (Statistics Denmark 2004).

ISCED=International Standard Classification of Education.

, and educational attainment was estimated for each level. Education higher than basic school was analysed in both unconditional analyses, that is, including all persons in both cohorts, and conditional on attainment of the previous educational level.

Person-years at risk were calculated from the age (in years) before the age of the first actual or possible attainment of each educational level: 13 years for basic school, 16 years for youth education, 18 years for short-, 20 years for medium- and 21 years for long-cycle higher education. All study subjects were followed until the level of education under investigation was attained or until death, emigration or the end of study period (31 December 2000), whichever came first. As persons could first be followed from 1980, we restricted the analyses to persons born after 1967 for basic school, 1964 for youth education and 1962 for short-cycle higher education. Thus, only the analyses of medium- and long-cycle higher education included all the birth cohorts. To ensure that the cancer and its treatment had a chance of influencing educational outcome, only patients whose cancer was diagnosed before possible attainment of a given educational level were included; thus, analysis of basic education was restricted to patients in whom cancer was diagnosed before they were 13 years of age. This approach is a modification of previous methods (Kelaghan *et al*, 1988; Haupt *et al*, 1994; Jensen *et al*, 1997).

### Data analyses

All multiple regression analyses included adjustment by current age and year of birth, and also for socioeconomic factors, comprising mean parental income (father's plus mother's annual income/2), highest educational level attained by either the father or the mother, number of biological siblings, whether the child lived with one or both biological parents or other new family combinations at the end of the year he or she turned 15, and the percentage unemployment experienced by the father during the current year. These adjustments did not alter the estimates significantly. Restriction of the analyses to persons who survived long enough to attain a given educational level, in order to investigate a possible effect of on-going disease, also showed no difference in educational pattern from that of all survivors.

Survival analyses, with calendar time as the underlying scale, were based on discrete-time Cox regression models, which facilitate time-varying covariates and offer the same interpretation of the estimated hazard ratio parameters as Cox models for continuous survival time (Allison, 1982; Breslow, 1992). The estimates given are rate ratios (RR) of attaining a given educational level. The models were fitted by the GENMOD procedure in SAS 8.2.

The study was approved by the Danish Data Protection Agency (Record # 2001-41-1354).

## RESULTS

### All cancers

Table 2

**Table 2:** Characteristics of cohort of survivors of childhood cancer

	**Men**		**Women**		**Total**	
**Characteristic**	**No.**	**%**	**No.**	**%**	**No.**	**%**
*Period of birth*	1367		1017		2384	
1960–65	313	23	208	20	521	22
1966–70	343	25	251	25	594	25
1971–75	376	28	312	31	688	29
1976–80	335	25	246	24	581	24
						
*Age at last year of follow-up (years)*						
13–19	170	12	129	13	299	13
20–24	395	29	277	27	672	28
25–29	346	25	276	27	622	26
30–34	269	20	193	19	462	19
35–39	187	14	142	14	329	14
						
*Age at diagnosis (years)*						
0–4 years	280	20	208	20	488	20
5–9	205	15	163	17	368	15
10–14	278	20	255	25	533	22
15–19	604	44	391	38	995	42
						
*Period of diagnosis*						
1960–69	94	7	52	5	146	6
1970–79	364	27	289	28	653	27
1980–89	598	44	478	47	1076	45
1990–96	311	23	198	19	509	21
						
Central nervous system tumours	**335**	**25**	**266**	**26**	**601**	**25**
						
Non-central nervous system tumours	**1032**	**75**	**751**	**74**	**1783**	**75**
Leukaemia	208	15	163	16	371	16
Lymphoma	235	17	123	12	358	15
Bone tumours	53	4	45	4	98	4
Gonadal neoplasms	202	15	46	5	248	10
Other solid tumours	334	24	374	37	708	30

shows the characteristics of eligible persons in the cancer cohort. Overall, 92% of cancer patients and 98% of controls had completed 9 years of compulsory basic education, corresponding to a RR of 0.90 (95% confidence interval (CI): 0.83–0.96) for all survivors of childhood cancer (both sexes). Youth education was completed by 60% of the cancer patients and 74% of controls, corresponding to a RR of 0.73 (95% CI: 0.68–0.78). Overall, equal proportions of cancer patients and controls attained some level of higher education (17 and 18%, respectively); however, these results cover differences by sex, as male survivors managed to complete all levels of higher education equally well as or even slightly better than control males (RR: 1.13, 95% CI: 0.97–1.32), whereas female cancer survivors had a reduced chance (0.75, 95% CI: 0.62–0.90).

### CNS tumours

Detailed regression analyses showed that survivors of non-CNS tumours completed most educational levels at a frequency and age similar to those of control persons (Table 3

**Table 3:** Rate ratio (RR) for survivors of childhood cancer attaining different educational levels, regardless of previous educational level, in comparison with the control cohort, by sex and diagnostic group

	**Men**					**Women**				
**Educational level**	**No. of eligible in each category^a^**	**Persons who completed education (%)**	**Median age at completion (years)**	**RR^b^ (95% CI)**	***P-*value**	**No. of eligible in each category^a^**	**Persons who completed education (%)**	**Median age at completion (years)**	**RR^b^ (95% CI)**	***P-*value**
*Level 1: Basic school*										
Controls	23 644	98	15.1	1		16 657	99	15.0	1	
Non-CNS tumours	355	92	15.0	0.97 (0.87–1.08)	NS	277	97	15.0	0.98 (0.86–1.10)	NS
CNS tumours	128	88	15.2	0.80 (0.66–0.96)	0.02	107	87	15.2	0.66 (0.54–0.81)	0.0001
										
*Level 2: Youth education*										
*Overall*										
Controls	27 629	72	19.2	1		19 413	77	18.9	1	
Non-CNS tumours	510	65	19.2	0.94 (0.84–1.05)	NS	429	69	18.9	0.85 (0.76–0.95)	0.005
CNS tumours	207	45	19.6	0.45 (0.37–0.55)	0.0001	172	42	18.9	0.40 (0.32–0.51)	0.0001
										
*Upper secondary school*										
Controls	27 629	34	18.5	1		19 413	51	18.5	1	
Non-CNS tumours	510	34	18.7	1.11 (0.96–1.28)	NS	429	48	18.5	0.92 (0.80–1.06)	NS
CNS tumours	207	21	18.8	0.48 (0.36–0.65)	0.0001	172	29	18.7	0.49 (0.37–0.64)	0.0001
										
*Vocational education*										
Controls	27 629	41	20.2	1		19 413	33	20.5	1	
Non-CNS tumours	510	34	20.4	0.84 (0.72–0.97)	0.02	429	28	20.8	0.89 (0.75–1.07)	NS
CNS tumours	207	28	20.4	0.68 (0.53–0.88)	0.004	172	19	20.9	0.56 (0.39–0.79)	0.001
										
*Level 3: Higher education*										
*Overall*										
Controls	29 460	16	24.3	1		20 783	21	24.4	1	
Non-CNS tumours	692	19	24.0	1.29 (1.09–1.54)	0.004	532	18	24.5	0.82 (0.67–1.01)	0.06
CNS tumours	264	13	24.7	0.77 (0.55–1.07)	NS	208	12	24.3	0.55 (0.37–0.82)	0.003
										
*Short-cycle*										
Controls	29 460	5	23.4	1		20 783	4	22.7	1	
Non-CNS tumours	692	5	23.4	1.22 (0.88–1.70)	NS	532	3	23.0	0.90 (0.57–1.44)	NS
CNS tumours	264	6	24.6	1.38 (0.85–2.27)	NS	208	1	22.8	0.40 (0.13–1.22)	NS
										
*Medium-cycle*										
Controls	29 456	10	24.2	1		20 895	17	24.6	1	
Non-CNS tumours	904	11	24.3	1.22 (1.00–1.48)	0.05	657	15	25.0	0.87 (0.71–1.06)	NS
CNS tumours	293	7	24.7	0.63 (0.41–0.98)	0.03	231	10	24.3	0.64 (0.43–0.95)	0.03
										
*Long-cycle*										
Controls	27 788	5	26.1	1		19 758	5	26.4	1	
Non-CNS tumours	846	5	26.0	1.12 (0.83–1.49)	NS	619	3	26.5	0.55 (0.35–0.88)	0.01
CNS tumours	273	4	25.9	0.56 (0.30–1.04)	0.07	214	4	26.9	0.89 (0.44–1.79)	NS

CI=confidence interval; NS=not significant at 5% level.

a Number of persons in each cohort who met the restrictions on year of birth and age at diagnosis, as described in Materials and Methods section.

b Adjusted for current age, birth cohort, mean parental income, parents' highest educational level, number of siblings, type of family of child at age 15 and father's degree of unemployment.

). In contrast, survivors of CNS tumours had reduced chances of attaining education at most levels, the RR of attaining all levels of higher education being 0.77 (95% CI: 0.55–1.07) for men and 0.55 (95% CI: 0.37–0.82) for women. When the analyses were conditioned on completion of youth education, no reduction in further educational attainment was found for any group of cancer survivors. In these analyses, male survivors of non-CNS tumours had a RR of 1.36 (95% CI: 1.14–1.61) and survivors of CNS tumours had a RR of 1.13 (95% CI: 0.81–1.57) of completing all levels of higher education, and the equivalent figures for women were 0.89 (95% CI: 0.72–1.09) for non-CNS tumours and 0.88 (95% CI: 0.59–1.32) for CNS tumours.

Characteristic differences in educational choices between diagnostic groups are also shown in Table 3. Male survivors of non-CNS tumours more frequently chose theoretical than practical education (vocational training and short-cycle higher education). In contrast, female survivors of non-CNS tumours had a smaller chance, of borderline significance, of completing upper secondary school than males (RR: 0.83, 95% CI: 0.68–1.01) and were more likely to complete short- and medium-cycle higher education than long-cycle higher education. Survivors of CNS tumours generally showed a preference for short, practical education, although the probability that the women in this group completed long-cycle higher education was close to normal. Subgroup analyses of survivors of CNS tumours showed that those who had survived infratentorial nonmedulloblastomas did not have a significantly reduced chance of attaining medium- or long-cycle higher education (RR: 0.72, 95% CI: 0.43–1.22, *n*=109), whereas the RR for survivors of other CNS tumours was 0.58 (95% CI: 0.42–0.79, *n*=415); however, these results did not differ significantly from each other.

Overall, childhood cancer survivors showed a significantly increasing trend in attaining youth education with increasing age at diagnosis (non-CNS tumours, RR per year: 1.02, 95% CI: 1.01–1.04, *P*-value=0.003; CNS tumours, RR per year: 1.06, 95% CI: 1.03–1.10, *P*-value=0.0004); however, this trend was not significant for higher education. Neither treatment era nor time since diagnosis appeared to have a strong influence on educational outcome.

During the decades investigated, the educational pattern in Denmark changed. Thus, multiple regression analyses of data for the control cohort showed a shift in educational choices over time, from vocational training to upper secondary school. Vocational education was most likely to be attained by persons from nuclear families, with one sibling and parents who earned middle incomes and had basic education or vocational training. The probability of being educated in general decreased with increasing number of siblings, degree of father's unemployment, living in a broken family and low parental income and education. These factors had similar effects in both cohorts.

The influence of social background, measured as the highest parental education, was tested in interaction regression analyses, where the pattern of the control cohort served as the standard. Survivors of CNS tumours whose parents had had higher education were more likely to finish vocational training than those whose parents had lower educational levels (RR 2.12, 95% CI: 1.36–3.31); however, male survivors of CNS tumours whose parents had higher education attained higher education less often than expected (RR 0.37, 95% CI: 0.18–0.78). Survivors of non-CNS tumours seemed more likely to attain long-cycle higher education if their parents had higher education than if the parents had had lower education (RR 1.61, 95% CI: 0.94–2.74).

## DISCUSSION

We found that survivors of non-CNS childhood cancers generally had a similar chance of attaining a given educational level as control persons and showed no substantial delay, whereas survivors of CNS tumours had a reduced level of educational attainment.

The socioeconomic and familial risk factors that influence educational attainment appeared to be similar for cancer survivors and controls, but female survivors showed more deficits in educational attainment than men. This might be due partly to general sex-specific differences in educational choices in Denmark, where vocational training and short-cycle higher education attract fewer women (Jensen *et al*, 1997; Statistics Denmark, 2004). Our results accord with those of others (Kelaghan *et al*, 1988; Langeveld *et al*, 2003; Mitby *et al*, 2003). Expectations of teachers with regard to academic performance might be lower for cancer patients and in conjunction with reports that girls are more often absent from school than boys (Charlton *et al*, 1991), this could indicate different cultural expectations for the two sexes.

Male survivors of CNS tumours tended to complete short, practical educational curricula rather than longer, theoretical ones. In contrast, female survivors of CNS tumours had a near-to-normal chance of attaining long-cycle higher education. CNS tumour survivors who completed upper secondary school showed no significant reduction in attainment of further education, and the chance of success increased with older age at diagnosis. These findings corroborate previous reports that young age at diagnosis is a risk factor for educational disadvantage (Kelaghan *et al*, 1988; Haupt *et al*, 1994; Langeveld *et al*, 2003; Mitby *et al*, 2003).

Cranial radiotherapy is known to be a strong risk factor for reduced educational attainment (Kelaghan *et al*, 1988; Haupt *et al*, 1994; Pastore *et al*, 2001; Langeveld *et al*, 2003; Mitby *et al*, 2003).This was reflected in our study by the better educational attainment level after infratentorial nonmedulloblastomas, probably treated with surgery only, in contrast to survivors of other brain tumours, who were more likely to have been treated with cranial radiotherapy. However, an internal comparison did not reach significance, which could be due to the analyses being based on small numbers in relation to this rather infrequent event. The finding that male survivors of CNS tumours had a reduced chance of completing basic education probably reflects the well-known risk for neuropsychological sequelae after cranial radiotherapy (Lannering *et al*, 1990; Jankovic *et al*, 1994; Langer *et al*, 2002; Reimers *et al*, 2003) and the subsequent learning difficulties, which cannot be compensated fully by special educational programmes (Peckham, 1991; Haupt *et al*, 1994; Dongen-Melman *et al*, 1997; Mitby *et al*, 2003). The finding that female survivors of CNS tumours had a reduced chance of attaining education at most levels may support the suggestion that the developing female brain is more vulnerable to cranial radiation (Schlieper *et al*, 1989; Bleyer *et al*, 1990). Deviation from the pattern of social heritage (Jensen *et al*, 1997; Lynch and Kaplan, 2000) only among survivors of CNS tumours also suggests a physiological rather than a psychosocial explanation for the reduced overall educational attainment.

Male survivors of non-CNS cancers seemed to do better than controls in attaining most educational levels and had a better chance of completing theoretical, longer education rather than shorter, technical courses. The reason is unclear. Previous studies showed that cancer survivors tend to undertake physical activity less than other persons (Mancini *et al*, 1989; Adamoli *et al*, 1997; Glaser *et al*, 1997) and that survivors of bone cancer with sequelae are more likely to have a high-school or college degree than those without sequelae (Pastore *et al*, 2001). The motivation of cancer survivors themselves, heightened awareness and support from parents or teachers (Peckham, 1991) or enrollment in special educational programmes (Mitby *et al*, 2003) might also play a role. Thus, in our study, support from well-educated parents tended to increase the probability of achieving the highest educational levels. Another study also found that the educational level of survivors of non-CNS tumours exceeded that of the general population (Pastore *et al*, 2001), and childhood cancer survivors have been described as more mature, with greater appreciation of parental support, than healthy controls (Maggiolini *et al*, 2000).

Our study confirms previous findings that only the survivors of childhood CNS tumours experience problems in achieving long-term educational outcomes (Kelaghan *et al*, 1988; Haupt *et al*, 1994; Pastore *et al*, 2001). The study of Langeveld *et al* (2003) accorded with our findings but lacked statistical power, which might explain the authors' more pessimistic conclusions of reduced educational attainment among cancer survivors in general, as also in another study (Teta *et al*, 1986). Mitby *et al* (2003) found educational deficits not only among survivors of CNS tumours and leukaemia, who had been treated with cranial radiotherapy, but also of non-Hodgkin's lymphoma and neuroblastoma. Their study was very large, but methodological limitations due to selection (only 12 431 of 20 276 eligible patients participated) and self-reporting of educational data may have influenced the results substantially.

To some extent, our study addressed these methodological problems. We included all cancer patients in Denmark, with no loss of follow-up or refusal to participate. All information on socioeconomic parameters, for both the cancer cohort and the randomly sampled control population, had been registered prospectively for administrative purposes, years before the current study was hypothesised, excluding information bias.

Our study also had limitations, however, including lack of treatment data and qualitative data that might have identified the mechanisms responsible for the educational patterns observed. Even though this was one of the largest cohort studies on educational attainment, some of our subanalyses lacked statistical power. In addition, not all persons could be followed to an age at which they were likely to have completed their education. Substantial delays during higher education could have underestimated ultimate educational attainments, especially in short- and long-cycle higher education. Future studies are required to identify interventions that might improve the outcome for survivors of CNS tumours.

## References

[bib1] Adamoli L, Deasy-Spinetta P, Corbetta A, Jankovic M, Lia R, Locati A, Fraschini D, Masera G, Spinetta JJ (1997) School functioning for the child with leukemia in continuous first remission: screening high-risk children. Pediatr Hematol Oncol 14: 121–131 9089740 10.3109/08880019709030898

[bib2] Allison PD (1982) Discrete-time methods for analysis of event histories. In Sociological Methodology, Leihardt S (ed) pp 61–98, San Fransisco, CA/London: Jorrey-Bass

[bib3] Berrino F, Gatta G, Sant M, Capocaccia R (2001) The EUROCARE study of survival of cancer patients in Europe: aims, current status, strengths and weaknesses. Eur J Cancer 37: 673–677 11311640 10.1016/s0959-8049(01)00008-9

[bib4] Birch JM, Marsden HB (1987) A classification scheme for childhood cancer. Int J Cancer 40: 620–624 3679589 10.1002/ijc.2910400508

[bib5] Bleyer WA, Fallavollita J, Robison L, Balsom W, Meadows A, Heyn R, Sitarz A, Ortega J, Miller D, Constine L (1990) Influence of age, sex, and concurrent intrathecal methotrexate therapy on intellectual function after cranial irradiation during childhood: a report from the Children's Cancer Study Group. Pediatr Hematol Oncol 7: 329–338 2268533 10.3109/08880019009033410

[bib6] Breslow N (1992) Use of the logistic and related models in longitudinal studies of chronic disease risk. Application: coronary heart disease mortality in Framingham. In Statistical Models for Longitudinal Studies of Health, Dwyer JH, Feinleib M, Lippert P, Hoffmeister H (eds). pp 163–196, New York/Oxford: Oxford University Press

[bib7] Charlton A, Larcombe IJ, Meller ST, Morris Jones PH, Mott MG, Potton MW, Tranmer MD, Walker JJ (1991) Absence from school related to cancer and other chronic conditions. Arch Dis Child 66: 1217–1222 1953006 10.1136/adc.66.10.1217 PMC1793478

[bib8] de Nully BP, Olsen JH, Hertz H, Carstensen B, Bautz A (1995) Trends in survival after childhood cancer in Denmark, 1943–87: a population-based study. Acta Paediatr 84: 316–324 7780256 10.1111/j.1651-2227.1995.tb13636.x

[bib9] Dongen-Melman JE, De Groot A, Van Dongen JJ, Verhulst FC, Hahlen K (1997) Cranial irradiation is the major cause of learning problems in children treated for leukemia and lymphoma: a comparative study. Leukemia 11: 1197–1200 9264369 10.1038/sj.leu.2400702

[bib10] Eiser C, Vance YH (2002) Implications of cancer for school attendance and behavior. Med Pediatr Oncol 38: 317–319 11979455 10.1002/mpo.1341

[bib11] Eurostat/Statistics Denmark (1995) Statistics on Persons in Denmark. A Register-based Statistical System. Brussels, Luxembourg: Office for Official Publications of the European Communities

[bib12] Glaser AW, Abdul Rashid NF, CL U, Walker DA (1997) School behaviour and health status after central nervous system tumours in childhood. Br J Cancer 76: 643–650 9303365 10.1038/bjc.1997.439 PMC2228016

[bib13] Haupt R, Fears TR, Robison LL, Mills JL, Nicholson HS, Zeltzer LK, Meadows AT, Byrne J (1994) Educational attainment in long-term survivors of childhood acute lymphoblastic leukemia. JAMA 272: 1427–1432 7933424

[bib14] Jankovic M, Brouwers P, Valsecchi MG, Van Veldhuizen A, Huisman J, Kamphuis R, Kingma A, Mor W, Dongen-Melman J, Ferronato L (1994) Association of 1800 cGy cranial irradiation with intellectual function in children with acute lymphoblastic leukaemia. ISPACC. International Study Group on Psychosocial Aspects of Childhood Cancer. Lancet 344: 224–227 7913156 10.1016/s0140-6736(94)92997-1

[bib15] Jensen TP, Mogensen KB, Holm A (1997) Valg og veje i ungdomsuddannelserne (Choices and Pathways in the Youth Educations). Copenhagen, Denmark: AKF forlaget

[bib16] Kelaghan J, Myers MH, Mulvihill JJ, Byrne J, Connelly RR, Austin DF, Strong LC, Meigs JW, Latourette HB, Holmes GF (1988) Educational achievement of long-term survivors of childhood and adolescent cancer. Med Pediatr Oncol 16: 320–326 3185360 10.1002/mpo.2950160506

[bib17] Koch SV, Kejs AMT, Engholm G, Møller H., Johansen C, Schmiegelow K (2004) Leaving home after cancer in childhood: a measure of functional independence in early adulthood, submitted 10.1002/pbc.20827 16572415

[bib18] Langer T, Martus P, Ottensmeier H, Hertzberg H, Beck JD, Meier W (2002) CNS late-effects after ALL therapy in childhood. Part III: neuropsychological performance in long-term survivors of childhood ALL: impairments of concentration, attention, and memory. Med Pediatr Oncol 38: 320–328 11979456 10.1002/mpo.10055

[bib19] Langeveld NE, Ubbink MC, Last BF, Grootenhuis MA, Voute PA, De Haan RJ (2003) Educational achievement, employment and living situation in long-term young adult survivors of childhood cancer in the Netherlands. Psychooncology 12: 213–225 12673806 10.1002/pon.628

[bib20] Lannering B, Marky I, Lundberg A, Olsson E (1990) Long-term sequelae after pediatric brain tumors: their effect on disability and quality of life. Med Pediatr Oncol 18: 304–310 2355890 10.1002/mpo.2950180410

[bib21] Lynch J, Kaplan G (2000) Socioeconomic position. In Social Epidemiology, Berkman LF, Kawachi I (eds). pp 13–35, New York: Oxford University Press

[bib22] Maggiolini A, Grassi R, Adamoli L, Corbetta A, Charmet GP, Provantini K, Fraschini D, Jankovic M, Lia R, Spinetta J, Masera G (2000) Self-image of adolescent survivors of long-term childhood leukemia. J Pediatr Hematol Oncol 22: 417–421 11037852 10.1097/00043426-200009000-00006

[bib23] Mancini AF, Rosito P, Canino R, Calzetti G, Di Caro A, Salmi S, Bonsi S, Marchi N, Paolucci G, Missiroli G (1989) School-related behavior in children with cancer. Pediatr Hematol Oncol 6: 145–154 2702068 10.3109/08880018909034280

[bib24] Mitby PA, Robison LL, Whitton JA, Zevon MA, Gibbs IC, Tersak JM, Meadows AT, Stovall M, Zeltzer LK, Mertens AC (2003) Utilization of special education services and educational attainment among long-term survivors of childhood cancer: a report from the Childhood Cancer Survivor Study. Cancer 97: 1115–1126 12569614 10.1002/cncr.11117

[bib25] Pastore G, Mosso ML, Magnani C, Luzzatto L, Bianchi M, Terracini B (2001) Physical impairment and social life goals among adult long-term survivors of childhood cancer: a population-based study from the childhood cancer registry of Piedmont, Italy. Tumori 87: 372–378 11989588 10.1177/030089160108700603

[bib26] Peckham VC (1991) Educational deficits in survivors of childhood cancer. Pediatrician 18: 25–31 1983857

[bib27] Reimers TS, Ehrenfels S, Mortensen EL, Schmiegelow M, Sonderkaer S, Carstensen H, Schmiegelow K, Muller J (2003) Cognitive deficits in long-term survivors of childhood brain tumors: identification of predictive factors. Med Pediatr Oncol 40: 26–34 12426683 10.1002/mpo.10211

[bib28] Schlieper AE, Esseltine DW, Tarshis E (1989) Cognitive function in long survivors of childhood acute lymphoblastic leukemia. Pediatr Hematol Oncol 6: 1–9 2641693 10.3109/08880018909014573

[bib29] Statistics Denmark (2004) Statistics Denmark: Yearbook 2002. Internet Communication: http://www.dst.dk/HomeUK/Statistics/Publications/Yearbook/2002.aspx → Education and culture pp1, date: 14-04-2004

[bib30] Statistics Denmark (1991) IDA – an Integrated Data base for Labour Market Research. Main Report. Copenhagen, Denmark: Statistics Denmark

[bib31] Storm HH, Michelsen EV, Clemmensen IH, Pihl J (1997) The Danish Cancer Registry – history, content, quality and use. Dan Med Bull 44: 535–539 9408738

[bib32] SU, The State Education Grant and Loan Scheme in Denmark (2004) Internet Communication: http://www.su.dk/in_english/default.htm date: 14-04-2004

[bib33] Teta MJ, Del Po MC, Kasl SV, Meigs JW, Myers MH, Mulvihill JJ (1986) Psychosocial consequences of childhood and adolescent cancer survival. J Chronic Dis 39: 751–759 3525599 10.1016/0021-9681(86)90158-x

[bib34] Thygesen L (1995) The register-based system of demographic and social statistics in Denmark – an overview. Stat J UN Econ Comm Eur 12: 49–55 12320272

